# Factors Facilitating and Hindering the Use of Newly Acquired Positioning Skills in Clinical Practice: A Longitudinal Survey

**DOI:** 10.3389/fmed.2022.863257

**Published:** 2022-05-04

**Authors:** Vera U. Ludwig, Heidrun Pickenbrock, Daniel A. Döppner

**Affiliations:** ^1^Department of Neuroscience, Perelman School of Medicine, University of Pennsylvania, Philadelphia, PA, United States; ^2^Wharton Neuroscience Initiative, University of Pennsylvania, Philadelphia, PA, United States; ^3^St. Mauritius Therapy Clinic, Meerbusch, Germany; ^4^Department of Information Systems and Information Management, University of Cologne, Cologne, Germany

**Keywords:** positioning, behavior change, skill acquisition, nursing, pressure ulcers, quality of care

## Abstract

**Background:**

After learning new skills, healthcare professionals do not always apply them in practice, despite being motivated. This may be referred to as an intention-behavior gap. One example is the positioning of immobilized and disabled patients in hospitals, nursing homes, or neurorehabilitation clinics. Positioning is crucial to prevent complications such as pressure sores, pneumonia, and deep vein thrombosis. However, it is often not carried out optimally even when professionals have completed education programs. The LiN-method is a positioning procedure involving a special focus on aligning and stabilizing body parts, which has been shown to have advantages over conventional positioning. We assess which factors may facilitate or hinder the use of LiN in clinical practice after participants complete training.

**Methods:**

A longitudinal survey with 101 LiN-course participants was conducted in Germany. Each participant completed a questionnaire directly after the course and 12 weeks later, including a report of the frequency of use in practice. They also completed a questionnaire which surveyed 23 aspects that might facilitate or hinder use of the new skills, covering the workplace, socio-collegial factors, motivation, self-confidence, and mindset.

**Results:**

Most assessed aspects were associated with LiN-use, with the highest correlations found for confidence with the method, perceived ease of application, sufficient time, assessing one's skills as sufficient, remembering the relevant steps, and a work environment open to advanced therapeutic concepts. To reduce data complexity, the questionnaire was subjected to a factor analysis, revealing six factors. A regression analysis showed that four factors predicted use 12 weeks after course completion, in the following order of importance: (1) subjective aspects/confidence, (2) access to materials, (3) work context, and (4) competent support in the workplace.

**Conclusion:**

Numerous aspects are associated with the use of recently acquired clinical or nursing skills, such as LiN. Many of these can be improved by appropriately setting up the workplace. The aspects most associated with use, however, are confidence with the method and self-perceived competence of healthcare professionals. While causality still needs to be demonstrated, this suggests that education programs should support participants in developing confidence and foster a mindset of continuous learning.

## Introduction

The intention-behavior gap is well-known and described in numerous domains, including the healthcare context (1, 2). It refers to the phenomenon that people have specific intentions for how they would like to act, but–often without understanding the reasons themselves– fail to act on these intentions. For example, nurses in hospitals know the importance of handwashing, yet often wash their hands less frequently than advised (3–5). Another important domain in the healthcare context is the positioning of severely affected immobile patients: Nurses, therapists, or caregivers often do not apply the positioning methods they learned frequently enough, or optimally, to prevent complications and enhance patient wellbeing (6–9). Determining why this is the case is crucial to derive recommendations on how to improve this situation.

To provide more background, numerous severely affected patients worldwide are unable to change their body position while hospitalized or being cared for at home. Reasons for immobility include stroke, dementia, or COVID-19 (10–12). Besides being discomforting for patients, this immobility can lead to complications such as pressure ulcers, deep vein thrombosis, pneumonia, and urinary tract infection (13–17). These factors can increase morbidity and mortality (18), hospital length of stay (19), and hospital costs (20, 21).

One way to address the risks of prolonged immobility is re-positioning patients in regular intervals (e.g., 2 h) (22, 23). This is frequently done in internal medicine, neurology, geriatrics, and intensive care units. The purpose of positioning is to prevent the aforementioned complications of prolonged immobility (24–26), to enhance patient comfort, and to improve the patient's respiratory condition (27). Positioning can also reduce the risk of aspiration, contractures, shoulder pain, and swelling (28, 29).

The LiN-method is a specific positioning concept developed around the year 2000. LiN is an abbreviation for the German term “Lagerung in Neutralstellung,” which translates to “Positioning in Neutral.” To regulate muscle tone and decrease the risk of contractures, the location of the body sections in relation to each other is analyzed and corrected in such a way that overstretching and shortening of muscles are avoided as much as possible. In contrast to conventional positioning, the body no longer adapts to the surface (often leading to malalignment, too much elongation, or shortening) and hollow spaces are avoided. The trunk and extremities are stabilized systematically with more blankets, bed covers, and pillows than usual to maintain the alignment of the whole body. Using more positioning material increases the supported areas, and the pressure on the body is thus lower in LiN-positions compared to conventional positions (30). In a randomized, multicenter, investigator-blinded, controlled trial, Pickenbrock et al. (31) compared the effects of LiN and conventional positioning on patient comfort and passive mobility. In 218 adult non-ambulatory patients with central neurological disorders, it was observed that patients perceived the LiN-positions as significantly more comfortable than the corresponding conventional positions. In addition, only in the LiN group, the passive mobility of the hips and shoulders improved when measured after 2 h of positioning. It was also shown that being positioned in LiN for 2 h had no negative effects on blood pressure, heart rate, and respiratory rate compared to being positioned conventionally (32).

However, despite the advantages of effective positioning methods, such as LiN, it is known that they are not always implemented in clinical practice. For example, nurses can often name the goals of positioning and different positions, but do not carry them out properly in practice (33–36). There is only scarce evidence on the effects of positioning training on clinical practice, but existing studies suggest that effects are often insufficient or even lacking (6, 7, 9).

It is currently unknown which specific factors contribute to the intention-behavior gap in positioning after completed training. We investigated potential reasons that might help or hinder healthcare professionals to regularly apply proper positioning methods in practice, using the example of the LiN-method. Conceivable are various aspects related to the workplace (e.g., accessibility of materials), colleagues' attitudes, study opportunities (e.g., refresher days), or confidence. Knowledge about the order of importance of these aspects may help to foster the use of effective practices, such as LiN, leading to benefits for patient health and enhancing the cost-effectiveness of courses offered to healthcare professionals. Therefore, the aim of this study was to assess how often LiN is applied in practice after course completion and which aspects may facilitate or hinder its application.

## Methods

### Study Design

We used a longitudinal survey design with two time points. After completing a LiN-course, participants were asked if they were interested in participating in the study. Participants then completed a questionnaire directly after course attendance and 12 weeks later.

### Questionnaire of Aspects of Potential Relevance to LiN-Use

A questionnaire about the reasons that might influence the frequency of LiN-use was developed in collaboration with a group of 40 LiN-trainers. All experts were asked for their opinion on which aspects might facilitate or hinder the use of LiN in clinical practice. Most provided their answers in written form, others were interviewed personally. After collecting and synthesizing the experts' opinions, the following three groups of possible reasons were identified:

- Workplace (8 items, e.g., “There is enough staff.”).
- Social factors/colleagues (10 items, e.g., “My colleagues approve of LiN and are cooperative with respect to its use.”).
- Motivation, confidence, and attitudes (5 items, e.g., “I am confident that I am doing everything correctly during LiN-application.”).

Each potential reason was formulated as a questionnaire item. The questionnaire did not directly ask whether participants believed that these aspects affected their LiN-use. Rather, participants rated the degree to which the items applied in their situation. As answer options, a Likert-scale was used (1: does not apply at all, 2: hardly applies, 3: partly applies, 4: mostly applies, 5. completely applies). The complete questionnaire can be found in Table 1 (see Supplementary Material 1 for the original German version).

**Table 1:** Questionnaire of aspects of potential relevance to LiN-use.

**Variable name**	**Item (translated from German)**
Sufficient Time	There is enough time for positioning.
Material Access	Material for positioning is easily accessible.
Material Location	Material for positioning remains where it is needed.
Storage Facilities	There is sufficient storage space for the materials.
Documentation	LiN-worksheets, posters, or info leaflets are within reach during the daily routine.
Practice	There is the opportunity to practice with less severely affected patients.
Enough Staff	There is enough staff.
Patient Stay Duration	Patients stay with us for long periods (>10 days).
Colleagues' Open-Mindedness	My colleagues show themselves to be open to novel therapy concepts.
Work Context Progressiveness	In my work environment, the appropriate context is being created for advanced therapeutic concepts and improvements of standard therapy.
Colleagues' Familiarity with Concept	Many of my colleagues are familiar with the concept of LiN.
Exchange with Colleagues	There is the opportunity to talk with colleagues who are also trained in LiN.
Colleagues' Advocacy	My colleagues approve of LiN and are cooperative with respect to its use.
Management Commitment	The management supports the concept of LiN.
Supervision	There is supervision of LiN during clinical routine.
Competence Team	There is a “LiN-competence team” on the ward.^*^
Education and Exposure	Lectures/educational events about LiN take place and/or there are flyers available regarding the positive effects of LiN, also for employees who have not participated in a course themselves.
Advanced Training	Participation in refresher days or advanced LiN-courses is made possible.
Self-Assessment	My knowledge and skills with respect to LiN seem sufficient to me for practical use.
Remember Steps	I remember the steps of the procedure or the positions for LiN-application.
Confidence	I am confident that I am doing everything correctly during LiN-application.
Ease of Application	The application of LiN appears to me easy and effortless.
Rating of Method	I feel that LiN is superior to conventional positioning.

*This is a translation of the original German version of the questionnaire (Supplementary Material 1). Variable names were not visible to participants*.

* *A “competence team” is a team experienced with LiN-use that is available to supervise others*.

### Variables

We collected the following variables of interest:

#### Time Point 1 (Within 1 Week After the Course)

- Estimate of the expected future LiN-use [in % of patients with LiN indication, measured in 10% steps using a sliding scale, similar to Herold and Kirsch (37)].
- Sociodemographics (age, gender, occupational group, years in the profession, etc.).

#### Time Point 2 (12 Weeks Later)

- Self-reported frequency of LiN-use (in % of patients with LiN indication, measured in 10% steps using a sliding scale).
- Questionnaire of aspects of potential relevance to LiN-use (see previous section).

The surveys also included some additional variables not analyzed here (see Supplementary Materials 2, 3).

### Sample Characteristics

Participants were included if the following criteria applied: (1) practices a caring profession (e.g., nurse, geriatric nurse, healthcare worker), or works as a physical therapist, occupational therapist, or in another therapeutic profession; (2) applies positioning in professional everyday life; and (3) is not a caring relative (i.e., positions exclusively privately). We had originally planned to include 200 participants based on a power analysis assuming relatively small effect sizes (i.e., pwr::pwr.r.test() in R showed that this sample size was required to detect correlations of r = 0.2 with a power of 0.80 and alpha = 0.05, two-tailed, when not correcting for multiple comparisons) (38). However, due to the COVID-19 pandemic, many courses were canceled, resulting in a smaller sample size.

### Survey Administration

The secure SoSci Survey questionnaire tool (39) was used for questionnaire implementation and data collection. Participants received links to the questionnaire via email as well as several reminder emails. Each participant had the chance to win one of six vouchers (2 x €50, 2 x €100, 2 x €150). Completing both questionnaires took a maximum of 35 min.

### Statistical Analysis

For the statistical analysis, the quantitative data was analyzed using Python 3.8 (40) with Pandas (41), Numpy (42), and Matplotlib (43), and R (44). The data were assessed for distribution and spread and then analyzed.

We used the following approach:

1. We calculated Spearman-rank correlations between each of the 23 aspects and actual LiN-use at time point 2. Due to the ordinal data structure, Spearman's rank correlations instead of Pearson's correlation coefficients were used. To avoid false positive findings, we corrected for multiple comparisons using the Holm-Bonferroni procedure (45, 46). We also estimated 95% confidence intervals for the correlation coefficients following the approach by Fieller, Hartley, and Pearson (47), using an R-script provided by Bishara and Hittner [Supplement A in (48)].
2. For a better intuitive understanding of the data, we also visualized how the top-half and bottom-half of LiN users differed in terms of the 23 aspects and demographics, by categorizing participants using a median split on the LiN-use variable. While it is difficult to define what constitutes objectively high or low use, a median split can give an approximate idea of the characteristics of participants using it relatively often vs. rarely.
3. Due to expected correlations amongst some of the 23 aspects, we applied factor analysis (FA) to reduce the questionnaire items to factor groups. FA is a multivariate statistical technique for reducing a large number of variables to fewer dimensions and is commonly applied in nursing research (49). We used exploratory rather than confirmatory FA in case our three predetermined categories were not accurate. To test the applicability of FA to our dataset we conducted Bartlett's test and the Kaiser-Meyer-Olkin (KMO) test (50– 52). For the FA, we used the FactorAnalyzer package for Python (53) with varimax rotation. We used an Eigenvalue threshold of 1.0 to extract factors. As recommended by Watson and Thomson (49), we describe the parameters of our FA as well as the resulting reduced dimensions.
4. The resulting factor scores from the FA were used as predictors in a multiple regression analysis with the outcome variable LiN-use at time point 2. For the estimation of the weighting of relationships between the variables, we used the ordinary least squares (OLS) method.

## Results

### Respondent Characteristics

From January to December 2020, 24 LiN basic courses were conducted in Germany by 14 different trainers (4 nurses, 7 physical therapists, 3 occupational therapists) with a total number of 230 course participants [mean: 9.58 participants per course, standard deviation (SD) = 2.448]. The typical length of a course was 2 days. 126 participants agreed to take part (55%). Out of these, 101 (92% female, mean age: 37 years, SD = 10.74, see Table 2 for more details) completed both questionnaires and were included in the data analysis (response rate: 80%).

**Table 2:** Demographics of the participants.

	**All participants** **(*****N*** **=** **101)**		**High application group** **(*****N*** **=** **50)**		**Low application group** **(*****N*** **=** **42)**	
	***N***	**%**	***N***	**%**	***N***	**%**
**Gender**						
Male	9	9%	3	6%	5	12%
Female	92	91%	47	94%	37	88%
**Age**						
21–39 years	36	36%	21	42%	9	21%
30–39 years	27	27%	13	26%	12	29%
40–49 years	20	20%	10	20%	9	21%
50–59 years	18	18%	6	12%	12	29%
**Working experience**						
<2 years	4	4%	2	4%	1	2%
2–5 years	28	28%	17	34%	7	17%
6–10 years	16	16%	9	18%	6	14%
11–20 years	26	26%	13	26%	11	26%
>20 years	27	27%	9	18%	17	40%
**Profession**						
Nurse	43	43%	21	42%	18	43%
Nurse assistant	0	0%	0	0%	0	0%
Geriatric nurse	11	11%	8	16%	2	5%
Geriatric nurse assistant	1	1%	1	2%	0	0%
Specialist nurse for intensive care medicine	7	7%	1	2%	6	14%
Occupational therapist	19	19%	7	14%	9	21%
Physiotherapist	17	17%	10	20%	6	14%
others	3	3%	2	4%	1	1%
**Workplace**						
Acute hospital	52	51%	23	46%	25	60%
Nursing home	5	5%	1	2%	4	9%
Neuro rehabilitation clinic	32	32%	20	40%	10	24%
Therapeutic practice	2	2%	1	2%	1	2%
Other	10	10%	5	10%	2	5%
**Discipline**						
Neurology	57	56%	32	64%	20	48%
Geriatrics	10	10%	2	4%	6	14%
Internal medicine	2	2%	1	2%	1	2%
Surgery	1	1%	1	2%	0	0%
Intensive care unit	11	11%	4	8%	7	17%
Intermediate care	2	2%	2	4%	0	0%
Others	18	18%	8	16%	8	19%

*For illustration purposes, data is also shown separately for a high LiN-use and a low LiN-use group, created by means of a median split. Nine participants who were exactly at the median were not included in either group, which explains why the overall sample size is larger than both groups combined*.

### Expected Use vs. Actual Use of the LiN-Method

Immediately after the course, participants expected to use LiN for a median of 60% of patients for whom LiN was appropriate. At follow-up, the median of the self-reported LiN-use was 40%.

### Correlations Between Questionnaire Items and LiN-Use

**Figure 1:**
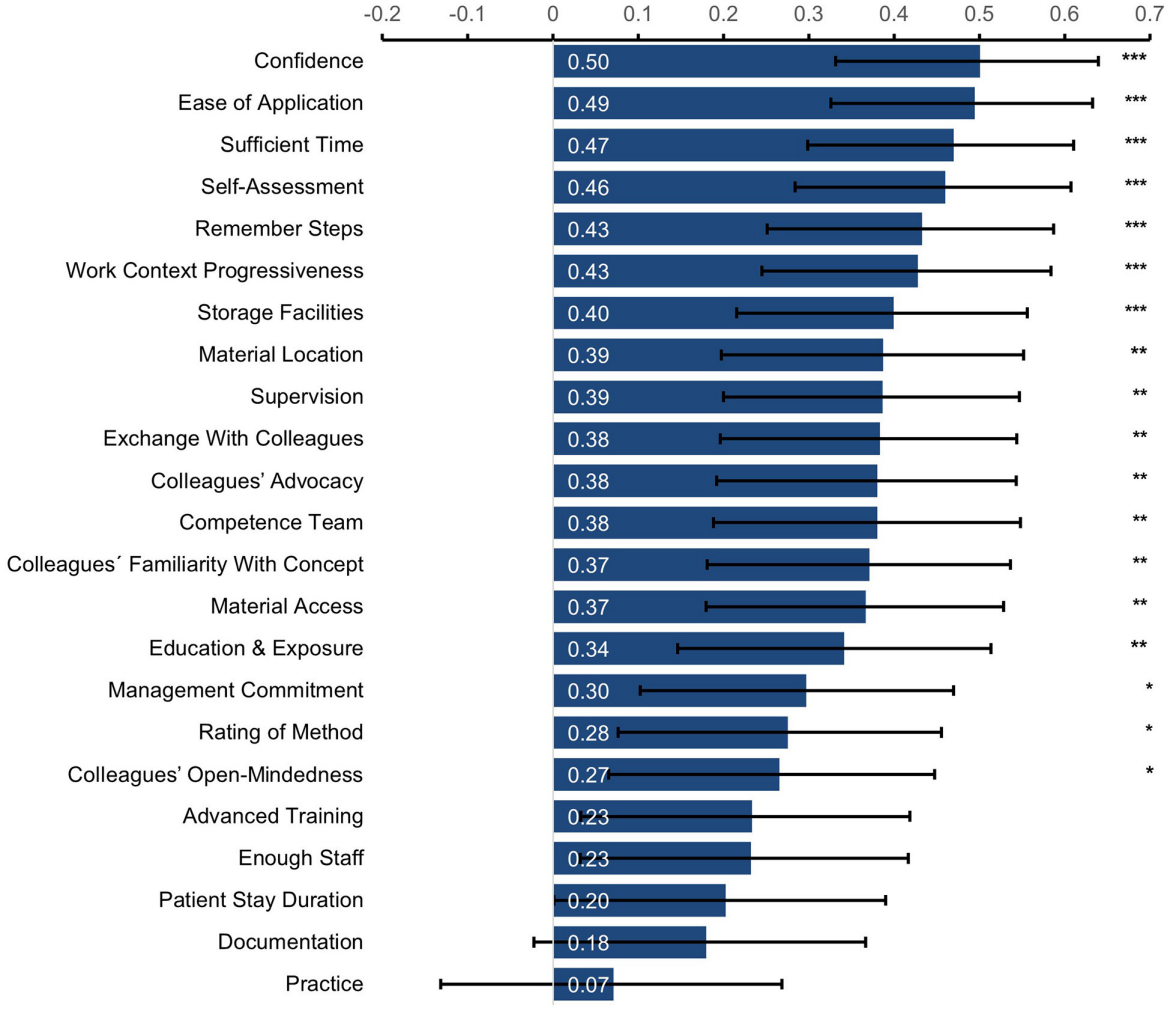
Spearman's rank correlation coefficients between self-reported LiN-use and the individual questionnaire items. Items are ordered by the size of the correlation coefficient. ***p < 0.001, **p < 0.01, *p < 0.05 (adjusted for multiple comparisons using Holm-Bonferroni correction). Error bars show 95% confidence intervals.

Figure 1 shows the correlation coefficients between all questionnaire items with LiN-use, ordered in descending order by the size of the coefficients. Most correlations were significant, even after correcting for multiple comparisons (54, 55). Of note, confidence intervals around the correlation coefficients were relatively large (Figure 1), leaving some uncertainty regarding the size of the effects.

### Ratings for Participants High and Low in LiN-Use

**Figure 2:**
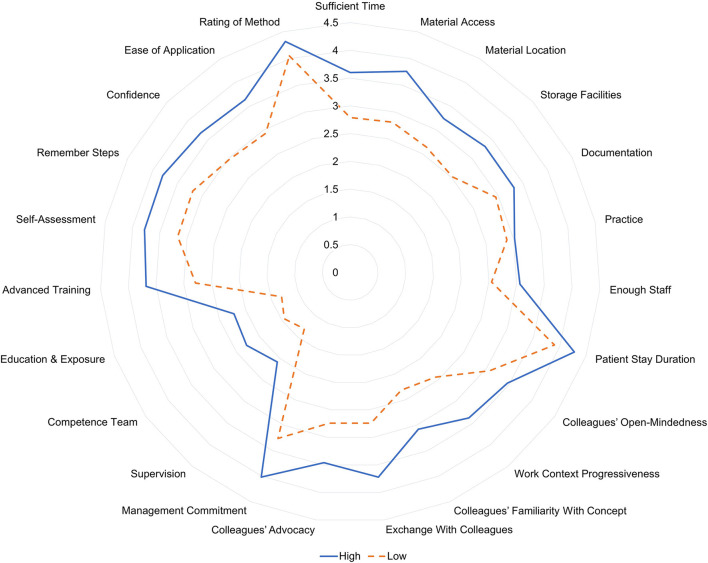
Comparison of item averages between the high LiN-use group and the low LiN-use group, derived by a median split.

To further visualize the data, we divided participants into two groups, applying a median split, resulting in a high LiN-use group (*N* = 50) and a low LiN-use group (*N* = 42) with a calculated median of 40% (nine participants with a score of 40% were not assigned to either group). Table 2 shows demographics for both groups. Figure 2 depicts average ratings on all questionnaire items and shows that all questionnaire items were rated as higher by the high-use group compared to the low-use group.

### Factor Analysis

FA is a statistical technique to reduce a high number of variables into fewer factors. It extracts maximum common variance from the individual variables and turns them into common scores. Despite a relatively small sample size, our analyses indicated that the data set was well-suited for FA (54): That is, the dataset had a KMO of 0.82 [with KMO value > 0.50 being considered acceptable (50, 51)], and Bartlett's Test of Sphericity (52) indicated that the observed correlation matrix was not an identity matrix (Chi-Square: 1082.78; *p* < 0.001).

The analysis procedure resulted in the selection of six factors. Table 3 shows how the items were grouped (indicated by bold font in each column). After reviewing the items contributing to each factor, we assigned the following six descriptive labels to the factors: 1. Subjective Aspects/Confidence, 2. Social Aspects, 3. Access to Materials, 4. Competent Support, 5. Training & Exposure, and 6. Work Context. The variance of the data explained by each factor ranged from 6 to 13% (see Table 3). All six factors together explained 56% of the variance.

**Table 3:** Results of the exploratory factor analysis including factor loadings and communalities.

**Variables**	**Loadings**						**Factor group**	**Communality**
**Labels assigned to factors (columns) and item labels (rows)**	**Factor 1** **Subjective aspects/** **confidence**	**Factor 2** **Social aspects**	**Factor 3** **Access to materials**	**Factor 4** **Competent support**	**Factor 5** **Training and exposure**	**Factor 6** **Work context**		
Self-Assessment	**0.792**	0.123	0.125	0.032	0.154	−0.078	1	0.690
Remember Steps	**0.755**	0.066	0.122	0.064	0.118	0.053	1	0.611
Confidence	**0.752**	0.160	0.185	0.147	0.040	0.122	1	0.665
Ease of Application	**0.668**	0.287	0.201	0.080	0.164	0.128	1	0.619
Rating of Method	**0.547**	0.182	−0.121	0.059	0.013	0.162	1	0.377
Colleagues' Advocacy	0.235	**0.735**	0.425	0.137	0.022	0.143	2	0.817
Colleagues' Open-mindedness	0.180	**0.634**	0.308	−0.060	0.070	0.176	2	0.571
Colleagues' Familiarity with Concept	0.256	**0.565**	0.477	0.315	0.109	−0.142	2	0.746
Management Commitment	0.194	**0.565**	0.008	0.281	0.188	0.361	2	0.603
Exchange with Colleagues	0.057	**0.500**	0.106	0.346	0.231	0.227	2	0.490
Practice	0.218	**0.370**	−0.119	−0.067	0.136	0.009	2	0.222
Storage Facilities	0.062	0.018	**0.728**	0.109	0.037	0.165	3	0.575
Material Location	0.111	0.121	**0.665**	0.004	0.171	0.262	3	0.568
Material Access	−0.050	0.125	**0.515**	0.077	0.073	0.327	3	0.402
Documentation	0.139	0.096	**0.351**	0.028	−0.078	0.000	3	0.159
Competence Team	0.085	0.057	0.076	**0.855**	0.139	0.015	4	0.767
Supervision	0.051	0.095	0.060	**0.820**	0.174	0.071	4	0.724
Patient Stay Duration	0.312	0.059	0.078	**0.356**	−0.010	−0.090	4	0.242
Education and Exposure	0.138	0.224	0.058	0.259	**0.921**	0.069	5	0.995
Advanced Training	0.163	0.122	0.032	0.098	**0.635**	−0.048	5	0.458
Work Context Progressiveness	0.218	0.455	0.208	0.175	0.190	**0.540**	6	0.657
Sufficient Time	0.291	0.107	0.303	0.023	−0.064	**0.517**	6	0.460
Enough Staff	−0.022	0.119	0.261	−0.065	−0.050	**0.499**	6	0.340
Eigenvalue	6.680	2.358	2.103	1.529	1.220	1.120		
Explained Variance Rate (%)	13%	11%	10%	9%	7%	6%		

*Items grouping together for each factor are printed in bold*.

### Regression Analysis

After reducing our data set to six factors, we entered these factors into a regression analysis to predict LiN-use. We found that Factor 1 (Subjective Aspects/Confidence), Factor 3 (Access to Materials), Factor 4 (Competent Support), and Factor 6 (Work Context) positively predicted LiN-use, while Factor 2 (Social Aspects) and Factor 5 (Training & Exposure) did not. The overall model including all factors explained 45% of variance in actual LiN-use (adjusted R-squared).

The unstandardized regressors shown in Table 4 correspond to the predicted increase in LiN-use on the original measurement scale, as predicted by 1SD increase for the respective factor score. For example, a 1SD increase in the factor score for Factor 1 (Subjective Aspects/Confidence) is predicted to be associated with an absolute increase of 14% in LiN-use (on a scale from 0 to 100% of patients for whom LiN-use would be appropriate). The standardized regressors are provided as well to allow comparison across studies. They reflect the predicted increase in LiN-use in SD-units, as predicted by 1SD increase in the factor scores.

**Table 4:** Results of ordinary least squares (OLS) regression analysis predicting LiN-use (in % of patients for whom LiN-use would be appropriate) based on factor scores.

**Variable**	**Unstandardized Coefficients (in % LiN-use)**	**Std. Error**	**Standardized Coefficients**	**t-Statistic**	**Prob**.
Constant	45.05	2.27		19.88	0.00^***^
Factor 1 (Subjective Aspects/ Confidence)	13.95	2.45	0.42	5.68	0.00^***^
Factor 2 (Social Aspects)	0.28	2.57	0.01	0.11	0.91
Factor 3 (Access to Materials)	11.03	2.61	0.32	4.22	0.00^***^
Factor 4 (Competent Support)	9.29	2.46	0.28	3.78	0.00^***^
Factor 5 (Training and Exposure)	3.98	2.27	0.13	1.75	0.08
Factor 6 (Work Context)	10.48	2.75	0.28	3.81	0.00^***^
R-squared	0.486				
Adj. R-squared	0.454				
F-statistic	14.84				
Prob (F-statistic)	0.000^***^				

*** *p < 0.001*.

## Discussion

After completing a course on the LiN positioning method, participants expected to use the newly acquired skills for a median of 60% of patients for whom LiN-use was considered appropriate. However, despite this high motivation, 3 months later they reported using it only for a median of 40% of patients. This study used a data-driven approach to identify aspects that might explain this intention-behavior gap, without having specific hypotheses about the relative importance of the various aspects. We found that the aspects most correlated with LiN-use were self-reported confidence with the method, ease of application, having sufficient time, assessing one's skills as sufficient, remembering the steps, and work context progressiveness (all correlations > 0.40, Figure 1). We also carried out a factor analysis of the 23 assessed aspects, revealing six factors (Table 3). In a linear regression, four of these factors predicted LiN-use (Table 4). The factor explaining the most variance was again the one related to subjective aspects/confidence with the method, followed by a second factor related to the availability and accessibility of sufficient materials. The third most important factor related to having sufficient time, staff, and a progressive work environment, and the fourth one comprised supervision and a “competence team” (i.e., experienced LiN-team that can give advice) at work.

These results are relevant because previous research has shown that quality of positioning in clinical practice is often poor, and training only helps to a limited extent. Lincoln et al. (34) observed that up to 13% of positioning on stroke units of a hospital in England was poorly performed, as well as 30% of positioning on other wards (medicine and geriatric). Dowsell et al. (35) report that patients with stroke spent 26% of their time in poor positioning. In terms of the effects of training, Jones et al. (9) showed that a 4-h training in positioning accompanied by a workbook led to 61% of all assessed aspects of posture to be done correctly, as opposed to 56% before the training [see also (6)]. While this was a significant increase, it is still not satisfactory. In a study by Forster et al. (7), after a 9-h training of positioning, 21% of patients still spent their time in poor positioning, compared to 31% before (a non-significant difference).

Our study suggests how the quality of positioning and of education programs might be improved by targeting certain aspects that are linked with the use of newly acquired positioning skills. Prominent theories of self-regulation and behavior change propose that, in order to regulate behavior (e.g., learn to apply a new nursing task regularly), one may modulate both external situation/environment (in this case: hospital environment and team context) and internal states (55). The current results suggest that for the use of newly acquired healthcare skills, both pathways might be important and both may–and perhaps must–be targeted to enhance the implementation of relevant practices after training.

Many environmental and team factors which were found to be important–such as offering sufficient storage capacity, enough personnel, etc.–should and can be provided by hospitals and organizations themselves. An important consideration here is that the costs for preventing pressure ulcers are much lower than treating the sores (56, 57). However, in addition to these external factors, our study shows that the aspects that correlated most with LiN-use were participants' internal states (e.g., confidence).

It is important to keep in mind that the current study was correlational and cannot speak to the direction of causality: For example, confidence with the method might lead to higher use, but higher use of the method might also increase one's confidence. Future studies should use interventions to target specific aspects identified here to determine if this would lead to higher use. It is also worth considering the potential clinical significance of the effects. Guidelines regarding the interpretation of correlation coefficients differ vastly and depend on context. However, some of the higher correlations found here (>0.40, Figure 1) would be classified by most guidelines as at least “moderate” in size (58–60); and they are higher than what is typically found in behavioral and psychological studies (61, 62). Other correlations, however, were small and all the confidence intervals (Figure 1) were relatively large, leaving uncertainty regarding the size of the effects (56, 57). The regression model further showed that each of the calculated factor scores predicted relatively substantial increases in LiN-use: A 1SD increase in Factor 1 (Subjective Aspects/ Confidence), Factor 3 (Access to Materials), Factor 4 (Competent Support), and Factor 6 (Work Context) predicted absolute increases in LiN-use of 14, 11, 9, and 10%, respectively. The links found here might be meaningful in practice, especially if they accumulate across people and time (61), but more research is needed to determine their significance.

How then can subjective factors such as confidence and ease of application be increased? One option might be to offer ample practice opportunities during training, offering encouragement, and–as proposed by Williams (63)–fostering a *growth mindset*. A growth mindset (64, 65) –as opposed to a fixed mindset–emphasizes that our talents are not set in stone, that we can learn new skills, and that mistakes are not a sign of weakness but rather useful for learning. A recent review (66) suggests that interventions to foster this mindset might be useful in health professions education [see also (67)]. Another possible intervention may be to direct healthcare professionals to pay attention to the positive consequences of using the new skills–such as being aware of the subjective feeling they experience when they help patients feel more comfortable (68).

The study has a few limitations. First, due to the COVID-19 pandemic, more than 50% of the planned LiN-courses were canceled. Therefore, we were only able to acquire a sample size that was smaller than initially planned, as is typical for many studies conducted in this time span (69). However, our sample size was still large enough to detect effects. For the factor analysis and regression analysis, the sample size was on the lower end, but appeared to still be acceptable (51, 70); for future use of the questionnaire, the factor structure should be reassessed in larger samples. Second, the COVID-19 pandemic led to changes in the work situation of health care professionals, such as increased pressure and psychosocial burden, which might have influenced our results (71–73) (see Supplementary Material 3 for some relevant comments by participants). However, even without the pandemic context, large intention-behavior gaps have been documented in many domains of human behavior change (2, 74). Third, there is a chance of a response bias: For example, it is possible that participants who opted in for the study were more motivated to apply LiN than those who did not. We cannot determine if such a bias was present in our sample, but the fact that almost 55% of course participants chose to take part in the study suggests that our results at least apply to a large proportion of potential course participants. Moreover, even in a sample of somewhat more motivated participants, it would still be relevant to determine which factors are associated with use after a course, since–as discussed–even highly motivated participants often apply new skills less than they planned.

The study also has several strengths. It is one of the first studies that systematically assesses a whole range of potential aspects that might influence the application of newly acquired clinical practices, including both internal and environmental aspects. Even though some of our results may seem unsurprising (e.g., access to materials correlates with higher use), the research confirming these intuitions was still lacking, and the relative importance of the various aspects was unknown. Moreover, we conducted a factor analysis to group the various aspects into factors predicting clinical practice. Previous research on educational programs for preventing pressure ulcers has been very limited (75). Finally, our results may not only apply to LiN but also to other newly acquired clinical skills or behaviors that are relevant in the clinical context, as well as to the design of effective training programs (76, 77).

In conclusion, we identified a set of aspects associated with LiN-use in practice months after course completion. The most important aspects appear to be participants' confidence and ease of application. However, there were indeed numerous aspects–relating to work set-up, colleagues, and motivation–that were clearly associated with higher LiN-use. This is hopeful news, as it suggests that by addressing many of these aspects simultaneously, a real difference can be made in terms of facilitating the use of newly acquired clinical skills. As LiN and other effective positioning methods can substantially improve patient mobility and wellbeing, as well as reduce risk for pressure ulcers and other complications, this new knowledge may be crucial for informing training approaches for healthcare professionals and, thus, for optimal clinical care.

## Data Availability Statement

The raw data supporting the conclusions of this article will be made available by the authors, without undue reservation.

## Ethics Statement

The studies involving human participants were reviewed and approved by Research Ethics Committee of the Chamber of Physicians Westfalen-Lippe and the Medical Faculty of the Westphalian Wilhelms University Muenster (2019-574-f-S). The patients/participants provided their written informed consent to participate in this study.

## Author Contributions

VL and HP designed the study, the questionnaire, and prepared the study materials. VL prepared the online questionnaire. All authors performed the data collection, with DD responsible for the technical implementation. DD analyzed the data and created the figures of results. All authors drafted, edited, provided feedback, and approved the final version.

## Funding

This study was funded by the LiN-Arge.

## Conflict of Interest

HP developed the LiN approach and is a member of LiN-Arge e.V., a non-profit organization that promotes the LiN approach. All authors have repeatedly received fees by LiN-Arge e.V. for working on research or other projects related to LiN in the past.

## Publisher's Note

All claims expressed in this article are solely those of the authors and do not necessarily represent those of their affiliated organizations, or those of the publisher, the editors and the reviewers. Any product that may be evaluated in this article, or claim that may be made by its manufacturer, is not guaranteed or endorsed by the publisher.
