# Supplementary material for: Factors Facilitating and Hindering the Use of Newly Acquired Positioning Skills in Clinical Practice: A Longitudinal Survey
Source: Front Med (Lausanne). 2022 May 4;9:863257. doi: 10.3389/fmed.2022.863257 (PMC9118333; doi:10.3389/fmed.2022.863257)
Supplement: Supplementary file 1 [file Data_Sheet_1.docx]

**Supplementary Material S1**

*Original German questionnaire to assess aspects of potential relevance to frequency of LiN-use*

1. Unsere Patient/innen haben einen langen Aufenthalt (>10 Tage) bei uns.
2. Es gibt die Möglichkeit, an weniger schwer betroffenen Patient/innen zu üben.
3. Lagerungsmaterial ist einfach zu besorgen.
4. Es gibt genügend Zeit zum Lagern.
5. Es ist genügend Personal auf der Station.
6. Arbeitsblätter, Poster und/oder Infoblätter zu LiN sind im Alltag in Reichweite.
7. Das Lagerungsmaterial bleibt an Ort und Stelle dort, wo es für die LiN-Lagerung benötigt wird.
8. Es gibt genügend Platz in den Zimmern, um das Lagerungsmaterial unterzubringen.
9. Kolleg/innen befürworten LiN und zeigen sich diesbezüglich kooperativ.
10. Es gibt die Möglichkeit zum Austausch mit Kolleg/innen, die auch in LiN geschult wurden.
11. Viele meiner Kolleg/innen kennen das Konzept der LiN-Lagerung.
12. Es gibt ein LiN-Kompetenzteam am Arbeitsplatz.
13. Es findet LiN-Supervision im klinischen Alltag statt.
14. Die Teilnahme an Refresher-Tagen oder dem LiN Aufbaukurs wird ermöglicht.
15. Das LiN-Konzept wird von der Leitung unterstützt.
16. In meinem Arbeitsumfeld wird der geeignete Rahmen für fortschrittliche Therapiekonzepte und Verbesserungen der Standardtherapien geschaffen.
17. Meine Kolleg/innen zeigen sich gegenüber neuartigen Therapiekonzepten aufgeschlossen.
18. Es finden Vorträge, Aufklärungsveranstaltungen statt bzw. gibt es Flyer zur Aufklärung über die positiven Effekte von LiN, auch für Mitarbeiter/innen, die selber keine Ausbildung absolviert haben.
19. Ich habe das Gefühl, dass die LiN-Lagerung der herkömmlichen Methode gegenüber überlegen ist.
20. Mein Wissen und Können in Bezug auf LiN erscheinen mir ausreichend für die Anwendung.
21. Ich kann mich gut an die Arbeitsschritte oder Positionen für die LiN-Anwendung erinnern.
22. Ich bin zuversichtlich, bei der LiN-Anwendung alles richtig zu machen.
23. Die Anwendung von LiN erscheint mir einfach und mühelos.

*Note. English translation of the items can be found in the main manuscript.*
