# Supplementary material for: Factors Facilitating and Hindering the Use of Newly Acquired Positioning Skills in Clinical Practice: A Longitudinal Survey
Source: Front Med (Lausanne). 2022 May 4;9:863257. doi: 10.3389/fmed.2022.863257 (PMC9118333; doi:10.3389/fmed.2022.863257)
Supplement: Supplementary file 2 [file Data_Sheet_2.docx]

**Supplementary Material S2**

*Other variables not analyzed in the manuscript*

Our overall survey included some additional questions that were outside the scope of the current article and were thus not further analyzed. These included questions about participants’ opinion about the LiN course and the usefulness of the method, details of participants’ work situation, the number of colleagues trained in LiN, the estimated required duration for LiN compared to CON, etc. Moreover, timepoint 2 included a voluntary essay question asking for why participants thought they used LiN less or more than intended. These comments were not further analyzed, but they can be found in the Supplementary Material S3.
