# Supplementary material for: Factors Facilitating and Hindering the Use of Newly Acquired Positioning Skills in Clinical Practice: A Longitudinal Survey
Source: Front Med (Lausanne). 2022 May 4;9:863257. doi: 10.3389/fmed.2022.863257 (PMC9118333; doi:10.3389/fmed.2022.863257)
Supplement: Supplementary file 3 [file Data_Sheet_3.docx]

**Supplementary Material S3**

*Participants’ comments regarding their reasons of using LiN more or less than planned*

At time point 2, participants answered the question “If you have used LiN less or more often than you originally thought or planned you would, why is that?”. The question was not obligatory, so not everyone answered. Comments were initially translated from German using www.DeepL.com/Translator (free version) and then translations were manually improved by author VUL. This list does not distinguish between participants who said they used the method less or more than they thought they would (evident by the content).

Comments:

- Sometimes limited in time or the patient's condition does not permit positioning at that moment. For example, sudden vomiting or fever.
- Through experiencing the comfort of LiN during the training myself, I am now more conscious about my positioning methods. It is not complicated, just as much effort as normal positioning. I have a bad conscience not to position according to LiN.
- Where I work has changed due to corona.
- Time, uncertainty, lack of understanding from others, you feel a bit smiled at with so much positioning material.
- Lack of time.
- I work 3 days a week as an art therapist; lack of time due to 30min treatment rhythm.
- I use LiN more often now, as because of my new work circumstances I have more time for the individual patient and I also have more positioning materials available.
- It’s due to the acceptance of the team that so much positioning material is used. Little understanding of the importance. We are working on this.
- My team is not trained and does not want to use it.
- I use LIN positioning regularly as it is more familiar to me now and I notice it is good for patients.
- High workload, lack of time, few positioning materials.
- Lack of cooperation with other professional groups.
- Practice is missing, colleagues position according to old patterns, do not want so much material in the bed.
- No routine, lack of supervision on the ward.
- Lack of time.
- We do not have time.
- I often lack the time to deal with this in more detail and practice.
- Covid 19, lack of working material.
- I think it is a great pity, as I find some of the LIN positions very good. At the moment, the percentage of patients for whom this would be appropriate is very low. Or in the last two weeks there were none at all. This means that the positioning options are quickly forgotten and you do not want to implement them incorrectly. In addition, I would find it nice if each of the colleagues were familiar with it, so that we can position uniformly. However, I plan to take the cards with me again and try to implement them again soon. Above all, I was a fan of the positioning in the sitting bed and in the chair.
- I am a role model for my colleagues as department head for neurological long-term rehabilitation.
- Due to the fact that too few employees are trained on the ward and this is then difficult to carry out so / to pull through. + Very high demand for positioning material, which is already calculated scarcely.
- Safe handling of concept since it has already been applied several times.
- Lin positioning was often not possible due to clinical picture. Residents partly did not tolerate.
- Since only I have had the positioning course, it is difficult to establish the positioning in everyday life of the colleagues. When I have positioned alone, I have usually used Lin positioning. I thought it would be easier to introduce the colleagues to the new way of positioning.
- At the moment the patients on the ward are positioned more by the nursing staff.
- Through Corona I could not get to my patients who were supposed to be positioned according to Lin. Before Corona I could position the patients twice.
- Currently work on wards with few positioning patients. Due to Corona pandemic no transfer to different wards.
- Not seen as suitable by many colleagues. Too many positioning materials. If I am then only part-time on site, it is very difficult to implement alone.
- Institution- positioning material.
- Rarely due to the change of wards.
- Corona.
- Few suitable patient, Covid-19- special situation.
- See above, change of working conditions due to Corona and an own disease with surgery.
- Corona- different patient clientele - all rather fit, no indication.
- Most of the patients feel very comfortable in the positioning.
- Too little positioning material, patient often on alternating pressure mattress.
- Current restructuring of patient clientele. Before we were a pure COVID ward.
- The many positioning materials that are needed. Time consuming.
- Stress, lack of staff.
- Due to the lack of routine for LIN positioning, conventional positioning is faster, although I try to implement the principles of LIN positioning.
- Extremely aggravated sick leave/staff shortage, therefore it is difficult to "try out”. In part, lack of acceptance by colleagues.
- Increasing staff shortage due to high sick leave and workload compression, leaving little time to try new things. Practically no colleagues who already know LiN and with whom I can exchange ideas.
- I have used the positioning much more frequently because I have achieved great success with it and I have subsequently saved myself work and gained time for other important things at the patient's bedside.
- It is largely the little time one has to perform LiN positioning. And it is made more difficult by the poor quality of the positioning materials. Especially the bad pillows for head alignment.
- Lack of material and willingness of colleagues to follow suit
- Time and material are usually too scarce in hospital operation.
- Most of the time I only apply one or two different LiN positions. This is partly because I am unsure of the other positions and partly because I always work alone.
- Used it more often. Especially after the new insights after the training.
- Practice makes perfect and it has helped sporadically.
- If I do not apply LiN, the reason is mainly that the nursing staff or other therapists have "just now" repositioned the patient. Then I optimize at least according to LiN principles everything that is still possible. If I get the task from the nursing staff to position the patient, I (intensive neuro rehab) exclusively use the LiN positioning.
- Missing positioning materials, time.
- often too little "material”; correct positioning according to LIN needs much time; with some patients would be nice to be able to work in pairs (it is partly also possible); lacks time to "only practice"; with the everyday stress one slips back to conventional.
- Due to a move fewer positioning material such as blankets, blankets also "bulkier".
- Mostly due to limited time or that the co-therapist or nurses have not done a LIN course.
- More time-consuming/ no material available and nursing is overburdened with a lot of positioning material/ or moving the patient back into other positions (e.g. from prone position).
- This has to do with my function as ward manager, I am currently more busy with documentation and tidying up the bureaucracy than with care at the patient's bedside.
- Too many patients with too few staff.
- I have noticed with the patients after LIN positioning that they sleep better in this position and lie more comfortably. Also the sitting in LIN position simplifies e.g. the food intake clearly.
- I can carry out Lin well alone. With Lin there were fewer complications with my Pat [patients] [with regards to] reddened support surface. Pat [patients] have felt more secure. Secure hold by Lin in Norbert [*might be the name of a hospital, unsure*] I personally found it very impressive, therefore I like to use it.
- Lack of time to learn the technique.
- Patient does not accept it.
- Applied it less than I thought: getting new positioning material every time, much forgotten by now about the positions.
- The knowledge that one can do better. Therefore one tries to do the positioning the best possible way. Otherwise one has a bad conscience not to have done the best possible.
- I only realized after the training that LIN is not suitable for every patient.
